# Quasi-experimental Effectiveness of Cognitive-behavioral Therapy on Reliving Migraine Headaches in Migraine Sufferers

**Published:** 2017

**Authors:** Hosein EBRAHIMI MOGHADAM, Ahoo KARIMI, Kimia SEIFI

**Affiliations:** 1 Department of Psychology, Roudehen Branch, Islamic Azad University, Roudehen, Iran.; 2 Young Researchers and Edite Club, Roudehen Branch, Islamic Azad University, Roudehen, Iran.

**Keywords:** Cognitive-behavioral therapy, Migraine headaches, Migraine sufferers

## Abstract

**Objective:**

This study aimed to investigate the effectiveness of cognitive-behavioral therapy on relieving migraine headaches in migraine sufferers.

**Materials & Methods:**

In this quasi-experimental study with pre-test and post-test method, the samples were outpatients of public hospitals in Ilam City, southwestern Iran since May-Jul 2010. They were selected based on inclusion and exclusion criteria, and divided into experimental and control groups. The data were analyzed using SPSS ver. 16 and via multivariate covariance method.

**Results:**

Cognitive-behavioral therapy affected on reducing the duration of symptoms of migraine in sufferers (*P*<0.05).

**Conclusion:**

Cognitive behavioral therapy effects on reducing the time duration of symptoms of migraine headaches. Thistherapeutic method increases the level of individual, familial, social and occupational activities by reducing the time duration of symptoms and this method helps the individuals to resume their activities and regain their previous control instead of founding themselves incapable and helpless due to the pain they suffer from.

## Introduction

Prevalence of migraine is different in various cultures, but it affects approximately 12% of general population and it is more effective on women. In various populations, the prevalence of migraine is about 6% in men and 18%of women aged between 15-45 yr old. Lifetime prevalence of migraine, is 8%-13% in women and 25%-33% in men (1).

With regard to occurring the migraine headaches after the stressful periods and the adverse effects of migraine headaches on psychological life of the sufferers and also the role of stress as the headache trigger, cognitive-behavioral therapy of headaches has been considered since 1970 (2). Over 50 years, improving many behavioral problems has been affected by cognitive-behavioral therapy. Behavioral therapy approaches developed in 1950 when empirical behavior basis was used to modify the behavior. Moreover, cognitive processes recognized and considered as an important treatment aspect in 1970 and finally it integrated with behavioral approaches named cognitive-behavioral therapy to improve psychological disorders (3). Over three decades, beneficial researches were conducted to reveal a number of behavioral therapy particularly Relaxation and Biofeedback and cognitive-behavioral therapy in the unpleasant form of tension and migraine headaches (4). 

The self-sufficiency on frequency, intensity, and in capability were relating to migraine among a sample of teens having migraine. The self-sufficiency is reversely related to the intensity and incapability of headache but it is not related to the frequency of headache. A research titled “A Meta-analytic review on pharmaceutical and behavioral therapies of migraine headaches” was performed and after studying 191 articles about the effectiveness of therapies on migraine headache, the whole tested therapies had similar effects and the level of effectiveness of all therapies on reducing migraine was 0.6-0.75.

Pain is usually temporary, but in some people, it persists over time -chronic pain. Chronic pain may lead to drug dependency, cause emotional distress and effect on people ability to engage in personal, social or recreational activities (5). Therefore, health care providers often prefer do not rely on chemical treatments and non-opioid analgesics to control long-term pains for two reasons: 1) Drugs often have undesirable side effects and may lead to psychological and physical dependency, 2) Chemical methods usually are insufficient to control pain and other approaches are required to help patients (6). Thus, the overall logic of cognitive-behavioral methods in treatment of migraine headaches stems from observing the ways that people cope with stresses of everyday life causing triggering, escalating or continuing of migraine attacks (2). 

Therefore, if the effectiveness of cognitive-behavioral therapy on migraine headaches is proved, it is possible for physicians, therapists, psychologists, psychiatrists to use this method as an appropriate guideline in order to improve supporting of patients with little side effects, and migraine sufferers receive the most benefit. Thus, this study aimed to examine the effectiveness of cognitive-behavioral therapy on reducing migraine headaches in sufferers. 

## Materials and Methods

The outpatients of public hospitals (Emam Khomeini &Taleghani) in IlamCity, southwestern Iran were examined since 2010. Out of 635 patients who were willing to cooperate, 30 migraine sufferers were selected regarding several factors: no pregnancy, lack of heart disease, minimum literacy and older than 15 yr old. These participants were divided into two experimental and control groups. The participants of experimental group received both drug and cognitive-behavioral therapy, and the participants of control group only received drug. The pre-test was conducted for both groups. The number of migraine attacks, duration of attacks, severity and the number of taking pain relief pills were controlled by phone within 4 wk. Eleven cognitive-behavioral therapy sessions were held for experimental group.

The study was approved by Ethics Committee of the university and informed consent was taken the patients.

The key issues trained in form of therapy sessions by the researcher included: Cognitive Restructuring (learning how to recognize the cognitive errors and replacing the positive and efficient thinking with negative and inefficient thinking), training the relaxation methods (abdominal breathing, imagination, progressive muscle relaxation), doing tasks and activities progressively regarding the time duration based on the resting/activity periods (without doing excessive activities ), assigning the home works in order to reduce the activities and resuming an active and effective life style.

Two participants of control group and one participant of experimental group discontinued the research processes. After the implementation of the independent variable, headache indicators (frequency, severity and the number of taking medicine) were measured within 8 wk. The results were analyzed using SPSS software (Chicago, IL, USA) and multivariate analysis of covariance.

## Results

Various descriptive indicators such as mean, standard deviation, and the Kolmogorov-Smirnov normality test results showed that the distribution of various scores tends to a normal distribution (regarding the score distribution of frequency, duration, and severity of headache symptoms and the degree of taking drug in post-test of both control and experimental groups after 8 wk) (Table 1)

**Table 1:** Summary of statistical indicators relevant to the scores of frequency, duration and severity of headache symptoms and the degree of taking drug in posttest of both control and experimental groups (N=27)

**Dependent variables**	**Variables**	**Stage**	**Mean**	**SD**	**K-s**	***P***
**Symptoms frequency**	Experimental	Post-test	4.07	1.154	0.121	0.169
	Control	Post-test	2.15	2.19	0.706	0.700
**Symptom duration**	Experimental	Post-test	33.07	25.14	0.682	0.741
	Control	Post-test	39.43	57.54	0.912	0.376
**Symptom severity**	Experimental	Post-test	27.07	18.89	0.801	0.543
	Control	Post-test	16.65	18.71	0.706	0.701
**Taking drug**	Experimental	Post-test	64.4	93.3	673.0	0.755
	Control	Post-test	61.9	86.22	350.1	052.0

**Table 2:** Summary of Lyon Variance Equality test

**Variables**	**F**	**Df** **1**	**Df** **2**	**Significant Probability**
**Symptom frequency**	0.895	1	25	0.363
**Symptom duration**	0.002	1	25	0.963
**Symptom severity**	0.990	1	25	0.329
**Taking drug**	3.262	1	25	0.083

**Table 3:** Summary of Multi-variable tests

**Effects**	**Tests**	**Value**	**F**	**Degrees of freedom**	**Error degree of freedom**	***P***	**Eta squared**
**Groups**	Wilkes Lambdatest	0.590	3.013	4	18	0.046	0.401

**Table 4:** Summary of effect tests onparticipants

**Resources**		**Ss**	**Df1**	**Df2**	**Ms**	**F**	**Eta squared**
Groups	Symptoms frequency	5.584	1	21	5.584	1.367	0.061
	Symptoms duration	4796.501	1	21	4796.501	* 4.429	0.174
	Symptoms severity	75.851	1	21	75.851	1.301	0.014
	Taking drug	218.389	1	21	218.389	0.724	0.033

To examine the effectiveness of cognitive-behavioral therapy on reducing the number of attacks, severity and duration of migraine headaches and the degree of taking drugs in migraine sufferers, Wilkes Lambda test was used, but at first, the precondition for the equality of variances using Lyon test was investigated in Table 2. Above assumption, of homogeneity of variance wasapproved regarding F value of Lyon variance equality test and significant probability in the four variables.

Regarding the value of Wilkes Lambda test (0.590) and F value (3.013) and degree of freedom of 18 & 4, there weresignificant difference between the mean of post-test scores in 4 variables (frequency, duration and severity headache symptoms and the degree of taking drug) in both control and experimental groups (*P*<0.05). According to Eta squared (0.401), the difference wasin moderate. Totally, Eta squared value (0.401) showed that there was an approximately strong relationship between cognitive-behavioral therapy and reducing the symptoms of migraine headaches (Table 3).

Calculated values with degree of freedom (1 & 21) showed that there wasno significant difference between the mean of post-test scores of control and experimental groups. However, there wasa significant difference between the mean of post-test scores of duration of headache symptoms in control and experimental groups (*P*<0.05). In respect of duration of headache symptoms, Eta squared value (0.174) showed an approximate weak relationship between cognitive-behavioral therapy and the duration of migraine headache symptoms (Table 4). 

## Discussion

In respect of the results of this research, cognitive-behavioral therapy effects on reducing the duration of headache symptoms in migraine sufferers. The results of this study are in consistent with another study(7). Another study showedrelatively small reduction of migraine headache indicators (with the exception of symptoms duration) (8). The results of another study showed that there was no significant difference between experimental groups, besides; there was recovery between experimental groups but not in control group within 6 weeks(9).Holroyd et al., conducted a study on 36 patients for 12-week treatment that 17 patients were taking drug (Amitriptyline in doses of 25, 50 and 75 mg) and 19 patients were treated by cognitive-behavioral therapy emphasizing on relaxation and problem-solving skills to cope with headaches(10). Both treatment methods were effective in reducing chronic headaches. However, cognitive-behavioral therapy has more effects on the improvement of life quality of the patients. In the current research also, cognitive-behavioral therapy wasrelatively effective in simultaneous reducing of post-scores of both control and experimental groups. Furthermore, according to self-report of the participants, cognitive-behavioral therapy led to decreasing damages due to headaches including withdrawal and sadness and learning more skills to cope with headaches. 

Therefore, long duration of migraine attacks have detrimental effects on the quality of individual and family life and career of the migraine sufferers as well.It affects on the ability and competence of people to perform social tasks because migraine sufferers have to rest somewhere dark and quiet to relieve headache pain (11). Moreover, inaction due to a headache causes increasing isolation, with draw a land depression (5). In the present study, there wasrelatively strong relationship between cognitive-behavioral therapy and reduction of symptoms duration of migraine headaches that lead to increasing individual, family, social and career activities of the patients. Furthermore, cognitive-behavioral therapy helps patients to restart their activities rather than feel disability due to their pain (5). However, cognitive-behavioral therapy instructs patients to change their dysfunctional and maladaptive thoughts. Psychologists and patients plan together to prevent and relieve the pain and compare this method of treatment with other types. It is more profitable onimproving weaker and non-psychoticdisorders (12). 

There were some limitations in this study such as refusing the patients to get involved in different stages of screening and researcher’s follow-up, age control, gender, and education level of patients and making limitations for generalizing the results and not increasing the number of samples due to the high cost of visiting neurologists. 

**In Conclusion, **all centers for counseling and psychotherapy, welfare, medical science, physicians, psychologists, consultants, psychotherapists, and neurologists should access to the results of the present study in order to provide better services, and reduce side effects in patients with migraine and tension headaches, and also prepare a therapist and patient handbook and deliver to the rated centers.
